# Commentary: Mobile phone dependence and musculoskeletal pain prevalence in adolescents: a cross-sectional study

**DOI:** 10.3389/fpain.2026.1781609

**Published:** 2026-04-08

**Authors:** Sergey Tereshchenko

**Affiliations:** Federal Research Center “Krasnoyarsk Science Center of the Siberian Branch of the Russian Academy of Sciences”, Research Institute of Medical Problems of the North, Krasnoyarsk, Russia

**Keywords:** adolescent, cell phone use, musculoskeletal pain, pain measurement, psychosocial factors

## 1 Introduction

The World Health Organization has recently highlighted a sharp rise in problematic digital device use among adolescents (1). Alongside well-documented mental health consequences, growing evidence links excessive smartphone and internet use to somatic symptoms, including headache and musculoskeletal pain (2). These associations warrant closer methodological scrutiny to distinguish correlation from causation and inform targeted prevention.

Parra-Fernandez et al. address a clinically meaningful and increasingly visible issue: the co-occurrence of adolescent musculoskeletal pain and mobile phone dependence (MPD) (3). In their school-based cross-sectional sample of 622 adolescents (10–18 years), more than half reported pain in at least one body region during the preceding six months, with upper back and neck pain being most prevalent. The authors also report higher MPD scores among adolescents reporting pain and identify the mobile phone “abuse and difficulty regulating use” dimension as significantly associated with general pain and neck pain in logistic regression models.

These findings are valuable for pain researchers because they move beyond screen time as a single exposure and instead examine dependence-related dimensions of device use. This commentary has three objectives: (1) to examine whether psychosocial factors may confound or mediate the reported MPD–pain associations; (2) to consider whether disaggregating device dependence by activity type could sharpen exposure assessment; and (3) to discuss how pain phenotyping and temporal alignment of measures could strengthen inference. These suggestions are offered to build on the contribution of the original study and to support comparability in future work.

### 1.1 Psychosocial factors: confounding and mediation

Parra-Fernández et al. appropriately note that the cross-sectional design prevents causal conclusions. This limitation is particularly relevant because psychosocial difficulties may plausibly act as (i) common causes of both dependence-like technology use and pain complaints, and/or (ii) intermediates linking dysregulated technology use to pain-related symptoms consistent with pathways involving stress and sleep disruption (4). Consequently, regression models that include age and sex but do not explicitly account for psychosocial factors may yield estimates that are difficult to interpret as “direct” effects of MPD on pain.

Validated instruments such as the Strengths and Difficulties Questionnaire (SDQ), the Pediatric Symptom Checklist, or the Youth Self-Report can efficiently capture psychosocial burden in school-based designs. Their inclusion materially changes effect estimates: in a large adolescent sample (*N* = 4,411), adding SDQ total scores as a confounder reduced odds ratios linking problematic internet use (PIU) to recurrent back pain by 22% and rendered the association with problematic video game use non-significant (4). Complementing this, mediation analyses with adults (*N* = 550) showed that PIU exerted no direct effect on neuropathic low back pain but significant indirect effects through depression and insomnia (5). These examples suggest that omitting psychosocial covariates may substantially inflate observed technology–pain associations, and that transparently reporting estimates with and without psychosocial adjustment would help readers gauge the robustness of MPD–pain links.

### 1.2 Clarifying the exposure: device dependence vs. activity type

A strength of the original article is its attention to MPD subdimensions, which is more informative than relying solely on an aggregate screen time metric. Nevertheless, “mobile phone dependence” remains a device-centered exposure that may aggregate heterogeneous internet behaviors (social media, messaging, gaming, streaming, school tasks) with potentially different links to pain through posture, repetitive strain, breaks, nighttime use, and arousal. This heterogeneity matters because an observed association could reflect a dominant activity pattern rather than dependence-like dysregulation *per se*. In a large-scale adolescent study, problematic social media use showed robust pain associations, whereas problematic video game use showed weak associations only with abdominal pain (4). This suggests that parsing device dependence by activity type and content may yield more specific and interpretable associations.

To improve interpretability, future studies could consider pairing an MPD scale with brief indicators of activity type and timing (e.g., evening/night use, dominant application categories) and then testing whether MPD dimensions remain associated with pain after accounting for these behavior-specific components. Related work using a “generalized vs. specific” approach to problematic technology use suggests that associations with pain-related outcomes may differ across specific forms of problematic use, reinforcing the value of parsing the exposure rather than treating it as a single construct. Such differentiation may help pinpoint the most actionable prevention targets (e.g., impaired control and late-night use, vs. total use).

### 1.3 Pain phenotyping and temporal alignment

Parra-Fernandez et al. use the Nordic Musculoskeletal Questionnaire and a six-month window to capture pain prevalence across body regions, which is a pragmatic and well-established approach for epidemiologic screening. However, dichotomous “pain yes/no” outcomes can combine brief, self-limited complaints with recurrent or functionally impairing pain, increasing heterogeneity and potentially diluting clinically meaningful signal. Where feasible, adding at least one severity dimension (frequency, intensity, or interference) would allow stronger clinical interpretation and facilitate comparisons with studies that define recurrent pain using combined frequency–intensity thresholds.

Temporal alignment between exposure and outcome windows is also important. If MPD captures relatively recent patterns while pain is assessed over six months, reverse causation (pain leading to altered device behavior) and recall issues may become more difficult to disentangle. Prospective follow-up or repeated short-window assessments (even if brief) could improve alignment and strengthen inference while remaining feasible in school settings.

## 2 Discussion

Parra-Fernandez et al. provide useful evidence that adolescent musculoskeletal pain co-occurs with higher MPD scores, and that the “abuse/difficulty regulating use” domain may be particularly relevant. The study therefore contributes to a growing pain literature that treats technology-related exposure as multidimensional rather than reducible to total screen time.

To further strengthen interpretation, future studies may benefit from prioritizing psychosocial measures, as key confounders/mediators, when modeling dependence-like constructs in relation to pain outcomes. Complementary refinements (differentiating device dependence from types and timing of digital activities, improving pain phenotyping beyond a binary outcome, and aligning exposure/outcome time windows) could help clarify mechanisms and inform more targeted prevention strategies for adolescents. Collectively, these methodological refinements are feasible within school-based designs and, if adopted, would enhance both the internal validity and cross-study comparability of findings in this rapidly growing research domain.
